# The Intersection of Human Rights, HIV and Sex Work: Implications for Ending AIDS in Thailand by 2030

**DOI:** 10.1002/jia2.70140

**Published:** 2026-07-01

**Authors:** Ravipa Vannakit, David Clarke, Surang Janyam, Chamrong Phaengnongyang, Chomnad Manopaiboon, Inthira Suya, Philippe Girault, Jintanat Ananworanich, Michael Cassell, R. Cameron Wolf

**Affiliations:** ^1^ Bangkok Interdisciplinary Research and Development Bangkok Thailand; ^2^ Amsterdam Public Health, Amsterdam University Medical Center, University of Amsterdam Amsterdam the Netherlands; ^3^ Amsterdam Institute for Global Health and Development Amsterdam the Netherlands; ^4^ Amsterdam Institute for Immunology and Infectious Diseases Amsterdam the Netherlands; ^5^ Service Workers In Group (SWING) Foundation Bangkok Thailand; ^6^ Independent Consultant Paris France; ^7^ FHI 360 Durham North Carolina USA; ^8^ AIDS Action Baltimore Maryland USA

**Keywords:** female sex workers, gender‐based violence, HIV/AIDS, human rights, key populations, male sex workers, PrEP, sex workers, Thailand, transgender women sex workers

## Abstract

**Introduction:**

Sex work significantly shapes the Thai economy and the HIV epidemic, yet its criminalization restricts access to health services and exacerbates human rights violations. This study examines experiences of stigma, discrimination, gender‐based violence (GBV) and incarceration among sex workers (SWs), exploring how these factors intersect with HIV services. This research is a component of a larger study investigating factors associated with pre‐exposure prophylaxis (PrEP) uptake among SWs in Thailand.

**Methods:**

We conducted a post hoc analysis of a cross‐sectional survey (September–December 2023) of 1511 Thai SWs recruited through convenience and quota sampling in seven provinces. Eligible participants were adults who self‐reported HIV‐negative or unknown status and had exchanged sex in the past 3 months. Trained peers administered anonymous face‐to‐face questionnaires covering demographics, work characteristics, HIV service utilization, stigma and discrimination, GBV, and PrEP. Descriptive statistics and bivariable and multivariable logistic regression were used to examine factors associated with past‐year human rights violations.

**Results:**

41.3% of SW participants reported experiencing at least one human rights violation, including stigma, discrimination, GBV and incarceration. Specifically, 18.1% experienced stigma and 35.8% reported GBV within the past year. Male sex workers (MSWs), transgender women sex workers (TGSWs), younger individuals, people who use drugs and those with a history of incarceration were the most affected groups. Geographical location was strongly correlated with these violations. A lack of human rights protections contributed to low uptake of HIV testing and sexually transmitted infection (STI) screening. PrEP uptake was critically low across all SW sub‐populations: 14.8% for TGSWs, 13.9% for MSWs and only 1.3% for female sex workers (FSWs).

**Conclusions:**

A human rights approach to HIV and sex work is indispensable to ending AIDS. Criminalization, stigma, discrimination and GBV intersect to produce layered vulnerability among different SW populations in Thailand, with TGSWs particularly affected. These structural conditions negatively shape access to HIV testing, STI screening and HIV prevention services, including PrEP. The findings reinforce that punitive legal environments and social stigma directly undermine HIV prevention efforts. Addressing the stigmatisation and human rights violations of sex workers are essential to ending AIDS in Thailand.

## 1 Introduction

A human rights‐based approach is considered essential to ending AIDS [1]. Countries with policy frameworks that promote human rights consistently achieve superior HIV testing and treatment outcomes compared to those utilizing coercive or punitive measures [2]. Rights‐based approaches are vital for creating enabling environments that affirm the dignity of people vulnerable to and living with HIV [3].

In Thailand, sex work is criminalized under the Prevention and Suppression of Prostitution Act (1996), which limits access to essential health services and exacerbates human rights violations against sex workers (SWs) [4], despite the historically significant role sex work has played in shaping the nation's economy and HIV landscape. Since the early 1990s, SWs have been central to Thailand's HIV prevention strategies. However, their legal and social marginalization has hindered the equity and sustainability of the response. Past attempts at epidemic control often prioritized controlling prostitution over improving SW human rights [5].

The national HIV prevention approach was anchored in the 100% Condom Use Programme, aimed at ensuring that clients of SWs could not purchase sexual services without condom use [6, 7]. The approach was primarily biomedical and enforcement‐driven, emphasizing surveillance, mandatory condom use and closure of non‐compliant venues. This approach neglected the underlying human rights issues that drive vulnerability and abuse.

Over the past decade, a more inclusive, community‐led model has emerged—the key population‐led health services (KPLHS) [8]. This initiative has strengthened trust, increased service uptake and shifted the narrative towards empowerment, participation and dignity. Yet, while Thailand's *National AIDS Strategy 2017–2030* commits to eliminating stigma, implementation gaps remain. Criminalization, police harassment, and a lack of social and occupational protections continue to undermine progress [3, 9, 10].

This study examines human rights violations among SWs in Thailand, focusing on stigma, discrimination and gender‐based violence (GBV). It explores how these experiences intersect with HIV prevention and is a component of a larger study investigating factors that limit pre‐exposure prophylaxis (PrEP) uptake among SWs across seven provinces [11]. Utilizing data from 1511 female sex workers (FSWs), male sex workers (MSWs) and transgender women sex workers (TGSWs), we investigate how sociodemographic and behavioural factors—including gender identity, age, HIV testing history, incarceration and drug use—are associated with human rights and health outcomes. These findings aim to inform evidence‐based, rights‐oriented policy development to support Thailand's goal of ending AIDS by 2030.

## 2 Methods

### 2.1 Study Design, Setting and Population

This post hoc analysis utilizes data from a quantitative cross‐sectional survey conducted between 26 September and 13 December 2023. The parent study [11] employed convenience sampling to recruit Thai SWs across seven provinces: Bangkok, Chonburi, Chiang Mai, Kanchanaburi, Phuket, Sa Kaeo and Udon Thani. Led by the SWING Foundation in partnership with Bangkok Interdisciplinary Research and Development (BIRD), the study collaborated with local community‐based organizations (CBOs) to access physical and virtual hotspots ensuring a diverse sample by reaching SW populations in provincial capitals where SWING did not have existing projects.

Participants were Thai national SWs from three groups: FSWs, MSWs and TGSWs. Eligibility required being aged ≥ 18 years, self‐reporting as HIV‐negative or of unknown status and having exchanged sexual services for money or goods in the past 3 months. Individuals self‐reporting an HIV‐positive status were excluded to ensure the sample represented the population for whom PrEP is clinically indicated.

### 2.2 Sampling Frame

Lacking reliable national size estimations for Thai SWs, the study used 2022 programmatic data from participating CBOs to determine sample size. In that year, approximately 18,820 SWs received HIV‐related services across the seven provinces. To achieve statistical significance, the target was set at 1500 participants, representing roughly 8% of the SWs reached previously. Quota sampling, informed by provincial CBO databases, ensured proportional representation of FSWs, MSWs and TGSWs. Within these quotas, SWING peer outreach workers used convenience sampling to recruit participants from physical and virtual hotspots. The final sample consisted of 1511 eligible participants. This slight over‐sampling ensured that the study maintained robust statistical power even after data cleaning and the exclusion of incomplete records.

### 2.3 Data Collection Tools

Trained peer outreach workers conducted face‐to‐face interviews using an anonymous, semi‐structured questionnaire entered into SurveyMonkey (SurveyMonkey Inc., San Mateo, California, USA). The instrument was adapted from validated WHO and UNAIDS tools, translated into Thai and piloted through SWING's outreach programme before final implementation. It utilized six components: (i) sociodemographics; (ii) occupational characteristics; (iii) health and HIV testing history; (iv) human rights violations (stigma, discrimination and GBV); (v) behavioural risks; and (vi) PrEP awareness and uptake. Human rights measures were grounded in UNAIDS Terminology Guidelines (2015) [12], defining HIV‐related stigma and discrimination as devaluation based on perceived or actual status. GBV was defined as any gender‐based act resulting in, or likely to result in, physical, sexual or psychological harm. Four GBV‐specific items were adapted from the WHO Multi‐Country Study and the Stigma Index to evaluate past‐year physical, emotional and sexual violence, and feelings of insecurity [13, 14]. SW experience of incarceration was investigated. The 12‐month recall period was used in alignment with WHO standards to balance data comparison with reliability.

### 2.4 Data Management and Statistical Analysis

Rigorous quality control included on‐site supervisor spot‐checks and continuous digital monitoring via SurveyMonkey validation rules. Following export, the dataset was imported into Stata (Release 15.1, 2017) for cleaning, de‐identification and review for inconsistencies or missing values to ensure integrity.

Variables were prepared through systematic recoding. Open‐ended responses were categorized by team consensus, and key demographics—such as age and education—were collapsed into binary variables. To maintain statistical power, locations with smaller samples (Kanchanaburi, Sa Kaeo and Udon Thani) were grouped into an “Other Sites” category. The primary dependent variable, reported human rights violations, was constructed as a binary measure: participants reporting any form of stigma, discrimination or GBV (physical, verbal, sexual or intimidation) were coded as 1, and all others as 0.

Data were analysed using descriptive and inferential methods in Stata (Release 15.1). Sample characteristics were summarized using frequencies and percentages for categorical variables, and means (SD) or medians (IQR) for continuous variables. Human rights violations were compared across populations using pairwise chi‐square tests with Bonferroni corrections. Complete‐case analysis was performed for regression modelling; however, alcohol use (15% missing) was excluded from these models due to a skip‐pattern error. Bivariable analyses used Pearson's chi‐square tests and logistic regression to calculate crude odds ratios with 95% confidence intervals. Variables with *p*≤0.20 were entered a multivariable logistic regression model to estimate adjusted odds ratios via manual backward elimination. Multicollinearity was assessed using a variance inflation factor threshold of ≥5, and model fit was evaluated using the Hosmer–Lemeshow test.

### 2.5 Ethical Considerations

The study protocol and instruments were approved by the Institute for the Development of Human Research Protections on 4 September 2023 (Protocol No. 103‐2566, COA No. IHRP2023114). Trained peer workers obtained verbal informed consent from all participants, ensuring they understood the study's voluntary nature and data confidentiality. Participants received 500 Thai Baht (∼15 USD) upon completion to compensate for their time and travel.

## 3 Results

### 3.1 Key Characteristics of the Participants

The sociodemographic characteristics of the study population are summarized in Table 1. Between 21 September and 11 December 2023, a total of 1511 SWs were interviewed, comprising 621 FSWs (41.1%), 452 MSWs (29.9%) and 438 TGSWs (29.0%). Recruitment was concentrated in major urban and tourist centres, with more than two‐thirds of respondents (70.0%) recruited from Bangkok, Pattaya and Phuket.

**TABLE 1:** Sociodemographic profile of respondents in the sex worker population.

	FSWs		MSWs		TGSWs		All	
	*N*	*%*	*N*	*%*	*N*	*%*	*N*	*%*
**Sites**								
Bangkok	120	19.3	104	23.0	101	23.1	325	21.5
Pattaya	137	22.1	104	23.0	151	34.5	392	25.9
Chiang Mai	102	16.4	98	21.7	62	14.2	262	17.3
Kanchanaburi	72	11.6	27	6.0	10	2.3	109	7.2
Phuket	140	22.5	102	22.6	98	22.4	340	22.5
Sa Kaeo	30	4.8	2	0.4	1	0.2	33	2.2
Udon Thani	20	3.2	15	3.3	15	3.4	50	3.3
*Total*	*621*	*100*	*452*	*100*	*438*	*100*	*1511*	*100*
**Age**								
18–24 years old	83	13.4	92	20.4	90	20.6	265	17.5
25–34 years old	184	29.6	202	44.7	197	45.0	583	38.6
35–44 years old	227	36.5	108	23.9	116	26.5	451	29.9
≥ 45 years old	127	20.5	50	11.1	35	8.0	212	14.0
*Total*	*621*	*100*	*452*	*100*	*438*	*100*	*1511*	*100*
**Level of education**								
Below secondary level	107	17.2	72	15.9	37	8.5	216	14.3
Secondary or above level	514	82.8	380	84.1	401	91.5	1295	85.7
*Total*	*621*	*100*	*452*	*100*	*438*	*100*	*1511*	*100*
**Partnership status**								
Single	392	63.1	304	67.3	351	80.1	1047	69.3
Married	52	8.4	12	2.6	—	—	64	4.2
With steady partner	177	28.5	136	30.1	87	19.9	400	26.5
*Total*	*621*	*100*	*452*	*100*	*438*	*100*	*1511*	*100*
**Have at least one child**								
No	206	33.2	380	84.1	435	99.3	1021	67.6
Yes	415	66.8	72	15.9	3	0.7	490	32.4
*Total*	*621*	*100*	*452*	*100*	*438*	*100*	*1511*	*100*
**Taking care of parents/relatives**								
No	141	22.7	122	27.0	76	17.3	339	22.4
Yes	480	77.3	330	73.0	362	82.7	1172	77.6
*Total*	*621*	*100*	*452*	*100*	*438*	*100*	*1511*	*100*
**Provide financial support for children and/or to parents**								
No	94	15.1	125	27.6	91	20.8	310	20.5
Yes	527	84.9	327	72.4	347	79.2	1201	79.5
*Total*	*621*	*100*	*452*	*100*	*438*	*100*	*1511*	*100*
**Additional occupation** ^a^								
None	550	88.6	335	74.1	375	85.6	1260	83.4
Student	2	0.3	7	1.6	—	—	9	0.6
Civil servant	1	0.2	—	—	—	—	1	0.1
Self‐employee	32	5.1	40	8.9	31	7.1	103	6.8
Employee in private sector	29	4.7	63	13.9	30	6.8	122	8.1
Day labourer	7	1.1	7	1.5	2	0.5	16	1.1
*Total*	*621*	*100*	*452*	*100*	*438*	*100*	*1511*	*100*
**Average monthly income** ^b^ **in THB**								
≤15,000 THB	138	22.2	151	33.4	75	17.1	364	24.1
15,001–25,000 THB	173	27.9	143	31.6	117	26.7	433	28.7
25,001–30,000 THB	101	16.3	67	14.8	88	20.1	256	16.9
> 30,000 THB	209	33.7	91	20.1	158	36.1	458	30.3
*Total*	*621*	*100*	*452*	*100*	*438*	*100*	*1511*	*100*
**Duration of sex work**								
≤ 6 months	121	19.5	62	13.7	54	12.3	237	15.7
> 6 and ≤ 12 months	128	20.6	89	19.7	88	20.1	305	20.2
> 1 and ≤ 5 years	197	31.7	170	37.6	131	29.9	498	33.0
> 5 and ≤ 10 years	105	16.9	80	17.7	77	17.6	262	17.3
> 10 years	70	12.3	51	11.3	88	20.1	209	13.8
*Total*	*621*	*100*	*452*	*100*	*438*	*100*	*1511*	*100*

Abbreviations: FSWs, female sex workers; MSWs, male sex workers; TGSWs, transgender women sex workers; THB, Thai baht currency.

^a^ In addition to sex work.

^b^ Income from sex work and, when applicable, from additional work.

Age distributions varied significantly across the three SW cohorts. FSWs were generally older, (median: 37 years; IQR: 28–43), while MSWs and TGSWs were notably younger, with median ages of 30 years (IQR: 26–38) and 31 years (IQR: 26–37), respectively. This age gap was further reflected in the youngest bracket (18–24 years), which accounted for approximately one‐fifth of both MSW and TGSW populations, compared to only 13.4% of FSWs. Regarding professional experience, nearly two‐thirds (64.1%) of all respondents had been engaged in sex work for more than 1 year. However, FSWs reported lower levels of long‐term experience (59.9%) than their MSW (66.6%) and TGSW (67.6%) counterparts.

### 3.2 Human Rights Violations: Stigma, Discrimination, GBV and Incarceration

As shown in Table 2, 18.1% of all respondents reported experiencing incidents of stigma or discrimination within the past 12 months. When stratified by population, TGSWs were significantly more likely to report such incidents (31%) than either MSWs (13.1%) or FSWs (12.7%), with both comparisons yielding high statistical significance (*p* < 0.001). In contrast, no statistically significant difference in reported stigma and discrimination was observed between FSWs and MSWs (12.7% vs. 13.1%; *p* = 0.291).

**TABLE 2:** Human rights violations among sex worker respondents.

	FSWs		MSWs		TGSWs		All	
	*N*	*%*	*N*	*%*	*n*	*%*	*n*	*%*
**Frequency of S&D**—**past 12 months**								
Never	542	87.3	393	86.9	302	69.0	1237	81.9
Rarely	52	8.4	45	10.0	95	21.7	192	12.7
Sometimes	20	3.2	12	2.7	26	5.9	58	3.8
Often	7	1.1	2	0.4	15	3.4	24	1.6
*Total*	*621*	*100*	*452*	*100*	*438*	*100*	*1511*	*100*
**S&D incident—past 12 months**								
No	542	87.3	393	86.9	302	69.0	1237	81.9
Yes	79	12.7	59	13.1	136	31.0	274	18.1
*Total*	*621*	*100*	*452*	*100*	*438*	*100*	*1511*	*100*
**Perpetrators of S&D**—**past 12 months** ^a^								
	*(N = 79)*		*(N = 59)*		*(N = 136)*		*(N = 274)*	
Client	32	40.5	19	32.2	59	43.4	110	40.1
Friend	15	19.0	18	30.5	19	14.0	52	19.0
Entertainment establishment staff	5	6.3	4	6.8	31	22.8	40	14.6
Unknown individual	7	8.9	6	10.2	20	14.7	33	12.0
Neighbour	15	19.0	8	13.6	8	5.88	31	11.3
Family member	7	8.9	2	3.4	6	4.4	15	5.5
Police officer	1	1.3	3	5.1	11	8.1	15	5.5
Healthcare provider	3	3.8	3	5.1	6	4.4	12	4.4
Spouse/steady partner	2	2.5	1	1.7	—	—	3	1.1
Social worker	—	—	—	—	—	—	—	—
**Physical violence—past 12 months**								
No	578	93.1	399	88.3	382	87.2	1359	89.9
Yes	43	6.9	53	11.7	54	12.3	150	9.9
Don't answer	—	—	—	—	2	0.5	2	0.1
*Total*	*621*	*100*	*452*	*100*	*438*	*100*	*1511*	*100*
**Verbal violence—past 12 months**								
No	506	81.5	359	79.4	302	69.0	1167	77.2
Yes	115	18.5	93	20.6	135	30.8	343	22.7
Don't answer	—	—	—	—	1	0.2	1	0.1
*Total*	*621*	*100*	*452*	*100*	*438*	*100*	*1511*	*100*
**Sexual violence—past 12 months**								
No	597	96.1	400	88.5	403	92.0	1400	92.6
Yes	24	3.9	52	11.5	34	7.8	110	7.3
Don't answer	—	—	—	—	1	0.2	1	0.1
*Total*	*621*	*100*	*452*	*100*	*438*	*100*	*1511*	*100*
**Intimidation—past 12 months**								
No	535	86.1	384	85.0	330	75.4	1249	82.7
Yes	85	13.7	68	15.0	107	24.4	260	17.2
Don't answer	1	0.2	—	—	1	0.2	2	0.1
*Total*	*621*	*100*	*452*	*100*	*438*	*100*	*1511*	*100*
**Any form of GBV**—**past 12 months**								
No	442	71.2	289	63.9	238	54.5	969	64.2
Yes	179	28.2	163	36.1	199	45.5	541	35.8
*Total*	*621*	*100*	*452*	*100*	*438*	*100*	*1511*	*100*
**Perpetrators of GBV**—**past 12 months** ^b^								
	*(N = 179)*		*(N=163)*		*(N = 199)*		*(N = 451)*	
Non‐regular client	69	38.6	59	36.2	90	45.2	218	40.3
Unknown individual	24	13.4	43	26.4	77	38.7	144	26.6
Friend	20	11.2	31	19.0	29	14.6	80	14.8
Regular client	5	2.8	7	4.3	21	10.6	33	6.1
Spouse/steady partner	19	10.6	10	6.1	4	2.0	33	6.1
Police officer	8	4.5	7	4.3	11	5.5	26	4.8
Family member	10	5.6	6	3.7	5	2.5	21	3.9
Neighbour	11	6.2	5	3.1	5	2.5	21	3.9
Don't want to answer	11	6.1	6	3.7	1	0.5	18	3.3
Entertainment establishment staff	3	1.7	2	1.2	10	5.0	15	2.8
**History of incarceration**								
Never	577	92.9	404	89.4	391	89.3	1372	90.8
In the past 12 months	21	3.4	12	2.7	18	4.1	51	3.4
More than 12 months ago	23	3.7	34	7.5	29	6.6	86	5.7
Don't want to answer	—	—	2	0.4	—	—	2	0.1
*Total*	*621*	*100*	*452*	*100*	*438*	*100*	*1511*	*100*

Abbreviations: FSWs, female sex workers; GBV, gender‐based violence; MSWs, male sex workers; S&D, stigma and discrimination because of working as a sex worker and/or sexual orientation; TGSWs, transgender women sex workers.

^a^ Multiple choice question among respondents who reported any S&D incident in the past 12 months.

^b^ Multiple choice question among respondents who reported any form of GBV in the past 12 months.

More than a third (35.8%) of all respondents reported experiencing at least one form of GBV—intimidation, physical, verbal or sexual violence—within the past year. TGSWs experienced the highest prevalence, being significantly more likely to report GBV incidents (45.5%) compared to both MSWs (36.1%); *p* < 0.01, and FSWs (28.2%); *p* < 0.001. Furthermore, a statistically significant difference was observed between the two cisgender groups, with MSWs reporting higher rates of GBV than FSWs (36.1% vs. 28.2%); *p* = 0.042.

Among the reported forms of GBV, verbal violence was the most prevalent (22.7%), followed by intimidation (17.2%), physical violence (9.9%) and sexual violence (7.3%). TGSWs were disproportionately affected by multiple categories of GBV: verbal violence (30.8%), intimidation (24.4%) and physical violence (12.3%). Notably, however, MSWs reported the highest prevalence of sexual violence (11.5%), surpassing both TGSWs (7.8%) and FSWs (3.9%). The most frequent perpetrators identified by respondents were non‐regular clients (40.3%), followed by unknown individuals (26.6%) and friends (14.8%).

Nearly one‐tenth of all respondents (9.1%) reported a history of incarceration, with 3.4% incarcerated within the past year and 5.7% more than 1 year prior. The prevalence of lifetime incarceration was notably higher among TGSWs (10.7%) and MSWs (10.2%) compared to FSWs (7.1%). These findings are particularly significant, given that incarceration is a documented driver of HIV transmission risk, due to restricted access to prevention tools and the prevalence of high‐risk behaviours within detention environments [2].

### 3.3 Sex Work and the Risk of HIV Acquisition

Behavioural risks for HIV acquisition are detailed in Table 3. In the week preceding the survey, 12.8% of MSWs, 10.7% of TGSWs and 10.5% of FSWs reported engaging in unprotected vaginal or anal sex. Group sex (sex with more than one partner simultaneously) within the past 3 months was reported by 29.7% of MSWs, 25.6% of TGSWs and 12.6% of FSWs. Crucially, the vast majority (95.1%) of those who engaged in group sex reported that their most recent encounter involved unprotected vaginal or anal intercourse.

**TABLE 3:** Behavioural risks for HIV acquisition and risk perception among sex workers.

	FSWs		MSWs		TGSWs		All	
	*n*	*%*	*n*	*%*	*N*	*%*	*N*	*%*
**Unprotected anal/vaginal sex with clients—past week**								
No	556	89.5	394	87.2	391	89.3	1343	88.7
Yes	65	10.5	58	12.8	47	10.7	170	11.3
*Total*	621	100	452	100	438	100	1511	100
**Participated in group sex—past 3 months**								
No	543	87.4	318	70.3	326	74.4	1187	78.6
Yes	78	12.6	134	29.7	112	25.6	324	21.4
*Total*	621	100	452	100	438	100	1511	100
**Any unprotected anal/vaginal sex at last group sex** ^a^								
No	3	3.8	11	8.2	1	0.9	15	4.6
Yes	75	96.2	122	91.0	111	99.1	308	95.1
Don't remember	—	—	1	0.8	—	—	1	0.3
*Total*	78	100	134	100	112	100	324	100
**Drug use before/during sex with clients—past 12 months**								
No	587	94.5	374	82.7	372	84.9	1333	88.2
Yes	34	5.5	78	17.3	66	15.1	178	11.8
*Total*	621	100	452	100	438	100	1511	100
**Frequent alcohol use before/during sex with clients—past 12 months**								
No	489	78.0	286	77.7	309	77.6	1084	77.8
Yes	115	22.0	82	22.3	89	22.4	286	22.2
*Total* ^b^	*524*	*100*	*368*	*100*	*398*	*100*	*1298*	*100*
**Medium to high HIV risk perception**								
No	340	54.7	255	56.4	214	48.9	809	53.5
Yes	280	45.3	197	43.6	224	51.1	702	46.5
*Total*	*621*	*100*	*452*	*100*	*438*	*100*	*1511*	*100*

Abbreviations: FSWs, female sex workers; HIV, human immunodeficiency virus; MSWs, male sex workers; TGSWs, transgender women sex workers.

^a^ Subsample: respondents who participated in group sex in the past 3 months.

^b^ Missing values (221 observations: 15% of total sample) due to technical issue with electronic data collection tools.

Drug use before or during sex with clients was reported by 11.8% of the total sample, with prevalence significantly higher among MSWs (17.3%) and TGSWs (15.1%) than FSWs (5.5%). The primary routes of administration were smoking (51.1%) and inhalation (41.0%), followed by swallowing (17.4%). Injection drug use was reported by 7.3% of the overall population. However, this was notably concentrated among MSWs (14.1%) compared to much lower rates in FSWs (2.9%) and TGSWs (1.5%). Additionally, 22.2% of respondents reported alcohol consumption before or during sex with clients, a trend consistent across all three sub‐populations.

Despite the high prevalence of documented risk behaviours, more than half of all respondents (53.5%) perceived themselves to be at low or no risk for HIV acquisition. This pattern of erroneous risk perception was remarkably consistent across all three sub‐populations, suggesting a widespread disconnect between actual exposure and subjective risk assessment within the community.

### 3.4 Correlates of Human Rights Violations

Overall, 41.3% of respondents reported experiencing at least one human rights violation in the 12 months preceding the survey. This prevalence was significantly higher among TGSWs (54.7%) compared to both MSWs (39.4%; *p* < 0.001) and FSWs (33.3%; *p* < 0.001), while no statistically significant difference was observed between FSWs and MSWs (33.3% vs. 39.4%; *p* = 0.131). Bivariable logistic regression confirmed that gender identity and SW sub‐population were strong predictors of violations, with MSWs and TGSWs showing stronger correlations than FSWs (see Table 4).

**TABLE 4:** Correlates of human rights violations among respondents in the sex worker population.

	Reported human rights incidents in the past 12 months						
	*Bivariable analysis (N = 1510)*					*Multivariable analysis* ^a^ *(N = 1508)*	
	*N*	*n (%)*	*p* value	*COR (95% CI)*	*p* value	*AOR (95% CI)*	*p* value
**Populations**			*p <0.001*				
Female sex workers	621	207 (33.3)		1		1	
Male sex workers	452	178 (39.4)		1.30 (1.01–1.67)	*p = 0.042*	1.01 (0.76–1.33)	*p = 0.962*
Transgender women sex workers	437	239 (54.7)		2.41 (1.88–3.10)	*p <0.001*	2.11 (1.61–2.77)	*p <0.001*
**Sites**			*p <0.001*				
Bangkok	325	161 (49.5)		1.78 (1.28–2.49)	*p = 0.001*	1.46 (1.02–2.09)	*p = 0.038*
Pattaya	391	151 (38.6)		1.1 (0.83–1.58)	*p = 0.419*	0.96 (0.68–1.36)	*p = 0.818*
Chiang Mai	262	93 (35.5)		1		1	
Phuket	340	126 (37.1)		1.07 (0.77–1.50)	*p = 0.693*	1.00 (0.70–1.43)	*p = 0.993*
Other sites^b^	192	93 (48.4)		1.7 (1.17–2.49)	*p = 0.001*	1.90 (1.27–2.85)	*p = 0.002*
**Age**			*p < 0.001*				
≤ 24 years old	265	140 (52.8)		1.76 (1.35–2.30)	*p < 0.001*	1.89 (1.41–2.53)	*p < 0.001*
> 24 years old	1245	484 (38.9)		1		1	
**Education level**			*p = 0.315*			*Excluded* ^c^	
Below secondary level	216	96 (44.4)		1.16 (0.87–1.55)	*p = 0.315*		
Secondary level or above	1294	528 (40.8)		1			
**Sex work duration**			*p = 0.277*			*Excluded*	
≤ 1 year	542	214 (39.5)		1			
> 1 year	968	410 (42.4)		1.13 (0.91–1.40)	*p = 0.277*		
**History of HIV testing**			*p = 0.002*				
Never been tested	172	52 (30.2)		1		1	
Ever been tested	1338	572 (42.8)		1.7 (1.22–2.43)	*p = 0.002*	1.72 (1.18–2.52)	*p = 0.005*
**History of STIs screening**			*p = 0.525*			Excluded	
Never been screened	731	296 (40.5)		1			
Ever been screened	779	328 (42.1)		1.07 (0.87–1.31)	*p = 0.525*		
**Currently using PrEP**			*p = 0.343*			Excluded	
No	1374	573 (41.7)		1.19 (0.83–1.71)	*p = 0.343*		
Yes	136	51 (37.5)		1			
**Any unprotected anal/vaginal sex with clients—past week**			*p = 0.200*				
No	1340	546 (40.8)		1			
Yes	170	78 (45.9)		1.1 (0.89–1.70)	*p = 0.200*		
**Participated in group sex—past 3 months**			*p < 0.001*				
No	1186	439 (37.0)		1		1	
Yes	324	185 (57.1)		2.26 (1.76–2.91)	*p < 0.001*	1.93 (1.47–2.53)	*p < 0.001*
**Drug use before/during sex with clients—past 12 months**			*p < 0.001*				
No	1332	505 (37.9)		1		1	
Yes	178	119 (66.9)		3.3 (2.37–4.60)	*p < 0.001*	2.66 (1.86–3.79)	*p < 0.001*
**Medium—High HIV perception**			*p = 0.012*				
No	808	310 (38.4)		1			
Yes	702	314 (44.7)		1.30 (1.06–1.60)	*p = 0.012*		
**History of incarceration** ^d^			*p < 0.001*				
Never	1371	539 (39.3)		1		1	
≤ 12 months	51	34 (66.7)		3.09 (1.70–5.58)	*p < 0.001*	2.54 (1.34–4.81)	*p = 0.004*
> 12 months	86	50 (58.1)		2.14 (1.38–3.34)	*p = 0.001*	1.68 (1.05–2.71)	*p = 0.031*

Abbreviations: AOR, adjusted odd ratio; CIs, confidence intervals; COR, crude odd ratio; HIV, human immunodeficiency virus; PrEP, pre‐exposure prophylaxis for HIV; STIs, sexually transmitted infections.

^a^ Goodness‐of‐fit test of the final model: *p* = 0.117.

^b^ Includes respondents from Kanchanaburi, Sa Kaeo and Udon Thani provinces.

^c^ Variable excluded from the initial logistic regression model as the *p*‐value > 0.20 in the bivariable analysis.

^d^ *N* = 1508: two respondents did not want to answer this question.

Several other variables demonstrated strong correlations with the experience of human rights violations. Geographic location played a significant role, with SWs in Bangkok, Kanchanaburi, Sa Kaeo and Udon Thani reporting higher violation rates than those in other surveyed sites. Age was also a critical factor, as younger SW aged 24 years or younger correlated more strongly with violations than older age ranges. Furthermore, behavioural and legal factors such as drug use before or during sex with clients in the past year and a lifetime history of incarceration were both strongly associated with an increased likelihood of experiencing violations compared to those without such histories.

Additional correlates emerged during the analysis that presented with lower face validity, suggesting a need for further investigation. These included: medium or high HIV risk perception, a history of HIV testing and participation in group sex within the past 3 months, all of which correlated more strongly with reported violations than their respective counterparts. Multivariate logistic regression confirmed the statistical significance of these correlations, potentially indicating that individuals who are more engaged with health services or more aware of their risks are also more likely to recognize and report abuse.

### 3.5 Barriers to HIV Testing, Sexually Transmitted Infections, Post‐Exposure Prophylaxis and PrEP Services

Across the sample, 64.9% reported healthcare access barriers, with consistent rates among MSWs (68.8%), FSWs (64.2%) and TGSWs (61.9%). Significant disparities existed by age and experience. SWs aged 18–24 were more likely to report barriers (71.3%) than older peers (63.6%; *p* = 0.016). Similarly, 69.6% of those in sex work ≤ 1 year reported barriers, versus 62.3% of those with >1 year experience (*p* = 0.005). Primary difficulties included lack of time (38.0%), unfriendly/stigmatizing services (13.5%) and financial constraints (10.3%).

Notably, 11.4% of SWs had never tested for HIV, peaking among FSWs (15.8%). SWs aged 18–24 were nearly three times as likely to have never tested (23.4%) as older counterparts (8.8%; *p* < 0.001). Likewise, 20.5% of those in sex work ≤ 1 year had never tested, compared to 6.3% of those with >1 year experience (*p* < 0.001). While 67.4% tested within the past year, the first‐year high‐risk window remains underserved.

Sexually transmitted infection (STI) screening mirrored HIV trends. However, overall STI engagement was lower; only 48.4% were ever screened. FSWs reported the highest lifetime screening (57.5%), followed by TGSWs (48.4%) and MSWs (42.0%). Despite low screening, 21.5% were treated for an STI within the past year (MSWs: 25.6%; FSWs: 24.2%; TGSWs: 14.6%), suggesting care‐seeking is symptomatic rather than preventive.

Post‐exposure prophylaxis (PEP) and PrEP utilization remained low. Only 7.6% had ever used PEP, driven by a 45.6% lack of awareness—a gap most pronounced among FSWs. PrEP was also underutilized (*p* < 0.001). While 14.8% of TGSWs and 13.9% of MSWs reported current use, FSW uptake was very low (1.3%). This underscores a disconnect between behavioural risks—especially unprotected group sex and substance use—and PEP/PrEP use (Table 5).

**TABLE 5:** Health service uptake among sex worker respondents.

	FSWs		MSWs		TGSWs		All	
	*N*	*%*	*n*	*%*	*n*	*%*	*N*	*%*
**Last HIV test**								
Less than 3 months	168	27.0	152	33.6	155	35.4	475	31.4
Between 3 and 5 months	153	24.6	104	23.0	91	20.8	348	23.0
Between 6 and 12 months	63	20.2	62	13.7	70	16.0	195	12.9
More than 12 months	139	22.4	89	19.7	93	21.2	321	21.2
Never been tested for HIV	98	15.8	45	10.0	29	6.6	172	11.4
*Total*	*621*	*100*	*452*	*100*	*438*	*100*	*1511*	*100*
**Ever been tested/screened for STIs**								
No	357	57.5	190	42.0	184	42.0	731	48.4
Yes	264	42.5	262	58.0	254	58.0	780	51.6
*Total*	*621*	*100*	*452*	*100*	*438*	*100*	*1511*	*100*
**Ever been diagnosed and treated for any STIs** ^a^								
Never	184	69.7	128	48.8	194	76.4	506	64.9
More than 1 year ago	16	6.1	67	25.6	23	9.1	106	13.6
Less than 1 year	64	24.2	67	25.6	37	14.6	168	21.5
*Total*	*264*	*100*	*262*	*100*	*254*	*100*	*780*	*100*
**Ever used PEP**								
I don't know PEP	33	53.6	179	39.6	177	40.4	689	45.6
Never used	274	44.1	233	51.5	200	45.7	707	46.8
Ever use	14	2.3	40	8.9	61	13.9	115	7.6
*Total*	*621*	*100*	*452*	*100*	*438*	*100*	*1511*	*100*
**Currently taking PrEP**								
No	613	98.7	389	86.1	373	85.2	1375	91.0
Yes	8	1.3	63	13.9	65	14.8	136	9.0
*Total*	*621*	*100*	*452*	*100*	*438*	*100*	*1511*	*100*
**Frequency of health services utilization when needed**								
Never	48	7.7	22	4.9	37	8.4	107	7.1
Some of the time	63	10.1	40	8.9	67	15.3	170	11.2
Most of the time	34	5.5	21	4.6	28	6.4	83	5.5
All the time	476	76.7	369	81.6	306	69.9	1151	76.2
*Total*	*621*	*100*	*452*	*100*	*438*	*100*	*1511*	*100*

Abbreviations: FSWs, female sex workers; HIV, human immunodeficiency virus; MSWs, male sex workers; PEP, post‐exposure prophylaxis for HIV; PrEP, pre‐exposure prophylaxis for HIV; STIs, sexually transmitted infections; TGSWs, transgender woman sex workers.

^a^ Subsample: respondents who reported ever been tested/screened for STIs.

## 4 Discussion

The study reveals a fragmented landscape of vulnerability where the burden of risk shifts based on gender identity, age and sex work experience. All three SW sub‐populations face an array of human rights violations in a context of criminalization of sex work with a systemic lack of legal protections. Stigma and discrimination are well evidenced in public health facilities in Thailand, often serving as a primary deterrent for SWs seeking HIV services [15]. These findings align with global evidence identifying punitive legal environments as the primary structural driver of HIV vulnerability among SWs [2, 16, 17].

TGSWs face the highest structural vulnerability, reporting the highest rates of nearly every human rights violation. This mirrors international reports where the intersection of transphobia and criminalization creates a double burden of marginalization, leading to disproportionate rates of violence and incarceration compared to cisgender peers [18, 19]. The disproportionate prevalence of stigma (31.0%), verbal abuse (30.8%), physical violence (12.3%) and human rights‐related intimidation (24.4%) suggests that transphobia, layered upon criminalization, creates a pervasively hostile environment where gender non‐conformity acts as a visible trigger for abuse. This double burden of marginalization not only increases exposure to violence but likely drives their 10.7% incarceration rate, the highest among surveyed SW populations.

MSWs emerged as a high‐risk group for severe physical and biological threats, reporting the highest rates of sexual violence (11.5%) and injection drug use (14.1%). This vulnerability is compounded by high‐risk sexual behaviours; MSWs reported the highest frequency of group sex (29.7%), of which 95.1% involved unprotected intercourse. Despite this intense risk profile, MSW PrEP uptake (13.9%) remains critically insufficient. The elevated rate of incarceration (10.2%) among MSWs further complicates this picture, as criminalization acts as a deterrent to seeking comprehensive HIV prevention services.

FSWs appear as an underserved population within the modern biomedical landscape. This reinforces a growing global concern that, as HIV programming pivots towards high‐risk male and transgender populations, cisgender women are being left behind by combination prevention strategies [11, 19]. While reporting lower rates of physical violence and incarceration than MSW or TGSW counterparts, they are the most left behind by contemporary prevention strategies. This is evidenced by a near‐zero PrEP uptake (1.3%) and the study's highest rate of never being tested for HIV (15.8%). The high rate of erroneous low‐risk perception (53.5%) among the total sample may be a primary driver for FSWs, who may perceive the traditional 100% Condom Strategy as sufficient, largely unaware of the added protection of PrEP. Consequently, national health strategies may be inadvertently deprioritizing cisgender women, leaving them without access to the full spectrum of combination prevention.

Across all cohorts, common vulnerabilities persist for younger SWs (<24 years) and those in their first year of work, who face the most significant barriers to care and the highest violation rates. These structural challenges are compounded by a pervasive risk‐perception gap; over half (53.5%) of the sample perceived themselves at low or no risk for HIV despite high rates of unprotected sex. This indicates a profound failure in current risk‐communication and outreach. Ultimately, addressing these disparities requires dismantling the structural barriers that equate sex work with criminality rather than a matter of public health and human rights. The risk‐uptake mismatch observed here is a documented challenge in many middle‐income settings [16], suggesting that current programming fails to address the specific needs of SWs navigating the complex intersections of substance use, group sex and legal precarity.

## 5 Conclusions

Ending the HIV epidemic among SW in Thailand requires not only a biomedically focused approach but also building a rights‐based structural intervention in a comprehensive HIV response. The study findings suggest that achieving National HIV Strategy goals is impossible without dismantling the stigma, discrimination and legal vulnerabilities that currently obstruct access to PrEP and other life‐saving health services. To bridge the observed risk‐uptake mismatch, Thailand must develop and implement a costed national strategy to enable the scaling up of PrEP options for each SW sub‐population. The current KPLHS model can be further optimized and adequately resourced to address the distinct health facility barriers faced by FSWs, MSWs and TGSWs. Crucially, KPLHS can be equipped beyond clinical care to provide comprehensive human rights protection and support, including dedicated services for GBV prevention and response, mental health, and legal and social welfare support. For these community‐led services to reach their full potential—particularly among the underserved FSW population and new entrants to sex work — they must be supported by an enabling legal environment that recognizes sex work as legitimate employment. Only by aligning biomedical innovation in a national strategic planning framework with robust human rights protections, sustainable financing, robust monitoring and evaluation and community leadership can Thailand close the HIV prevention gap and ensure the safety and health of all SWs—MSWs, FSWs and TGSWs, and their clients.

This study is not without limitations. Firstly, the use of convenience sampling for site selection and recruitment by CBOs introduces potential selection bias as well as potential social desirability biases. The non‐probability approach may over‐represent SWs already reached by service networks, potentially limiting generalizability to those outside formal community support. Furthermore, the cross‐sectional design precludes the establishment of temporal or causal relationships between human rights violations and HIV risk behaviours. Second, the study did not utilize multidimensional validated tools, such as the Berger HIV Stigma Scale, nor did it assess mental health outcomes—including depression and post‐trauma distress disorder—which are critically linked to stigma and GBV. Finally, the exclusion of people living with HIV from the parent study likely results in an underestimation of the true burden of stigma and discrimination, as SWs living with HIV often face layered vulnerabilities and heightened rights violations. Despite these constraints, the large sample size across three distinct populations provides a robust snapshot of the current human rights landscape for SWs in Thailand.

## Author Contributions

Conceptualization, methodology and project administration: SJ, CP and RV; Data collection and analysis: SJ, RV, CP and CM; Writing of the manuscript: RV, DC, PG and IS; Review and editing: CPG, MC, CW and JA. All authors have read and agreed to the published version of the manuscript.

## Funding

The study was funded by Gilead Sciences, Inc. The findings and conclusions in this report are those of the authors and do not necessarily represent the views of Gilead Sciences, Inc. The funder had no role in study design, data collection and analysis, decision to publish or preparation of the manuscript.

## Conflicts of Interest

The authors declare no conflicts of interest.

## Data Availability

The data that support the findings of this study are available on request from the corresponding author. The data are not publicly available due to privacy or ethical restrictions.
